# Impact of Anaerobic Pyrolysis Temperature on the Formation of Volatile Hydrocarbons in Wheat Straw

**DOI:** 10.3390/ma19020436

**Published:** 2026-01-22

**Authors:** Kamil Roman, Dominika Szadkowska, Jan Szadkowski

**Affiliations:** 1 Department of Technology and Entrepreneurship in the Wood Industry, Institute of Wood Sciences and Furniture, Warsaw University of Life Sciences, 166 Nowoursynowska St., 02-787 Warsaw, Poland; kamil_roman@sggw.edu.pl; 2 Department of Wood Science and Wood Protection, Institute of Wood Sciences and Furniture, Warsaw University of Life Sciences, 166 Nowoursynowska St., 02-787 Warsaw, Poland; jan_szadkowski@sggw.edu.pl

**Keywords:** pyrolysis, biomass, straw, PY/GC-MS, wheat straw

## Abstract

The anaerobic thermal decomposition of plant biomass produces raw materials such as wood charcoal, wood oil, or biogas, which can be used to replace conventional fossil fuels. This enables the development of environmentally friendly alternatives to traditional fuels without the need to develop new technologies, such as engines. The aim of the study was to verify the substances produced during the anaerobic thermal decomposition process of wheat straw. Measurement was carried out by pyrolysis at eight selected temperatures between 350 °C and 1050 °C, with an increase of 100 °C. The analysis was performed on a pyrolyzer coupled to a gas chromatograph (PY/GC-MS). An ANOVA test was used to detect the significance of the results. Based on the ANOVA analysis, the distribution of compound classes in the three temperature regimes was statistically significant. Phenolic compounds reached their highest relative abundance (or relative content) at 650 °C, while PAHs (polycyclic aromatic hydrocarbons) were absent below 550 °C and increased sharply above 850 °C. The results illustrate the thermal decomposition pathway of straw biomass: low-temperature pyrolysis favors the formation of oxygen-rich bio-oils, while higher temperatures increase aromatic condensation and PAH production.

## 1. Introduction

Thermal decomposition of lignocellulosic biomass is the traditional method of obtaining raw materials such as charcoal, wood tar, and wood gas [1,2,3]. These substances were used in energy, industry, or everyday life. A characteristic feature of the production of these products was the thermal decomposition without air of the lignocellulosic biomass. The quality and composition of the pyrolysis products obtained depends on the temperature used and the pressure during the thermal decomposition process [4,5,6]. The methods of this convention have lost relevance as a result of the discovery and development of the use of fossil fuels such as coal, petroleum oil, and natural gas. These raw materials were simpler to process and use in industry [7,8].

The discovery and detection of the greenhouse effect and the impact of industry on its intensification have contributed to the development of research work focused on finding alternative sources of energy and fuels to fossil raw materials. One direction of development for energy raw materials is the production of biofuels. In order to obtain them, a return to traditional methods of biomass conversion is indicated, with the application of knowledge and modern developments, such as optimization of process control, increasing reaction pressure, use of reaction catalysts, etc. [9,10,11,12].

Biomass in agriculture, such as straw, is the residue that has the potential to be converted into sustainable bioenergy. Bio-oil, gas fuels, and charcoal can be produced by thermochemically converting straw via pyrolysis, such that pyrolysis can produce renewable energy and charcoal as a soil amendment. Temperature plays an essential role in determining the chemical composition of pyrolysis products [13]. As the pyrolysis temperature is lowered, oxygenated organic compounds (including acids, furans, and phenolics) are generated from cellulose, hemicellulose, and lignin. The higher the temperature, the more deoxygenation and polycondensation occur, and the more aromatic structures are formed [14,15]. To optimize bio-oil quality (energy content, stability) and minimize toxic products such as polycyclic aromatic hydrocarbons (PAHs), it is essential to understand how the straw’s volatile product spectrum changes with temperature.

Polysaccharides such as cellulose and hemicelluloses are a construction part of straw, and polymers like lignin, which is also referred to as phenylpropanoid polymer [16]. Minor proteins and extracts contribute to the straw structure. Different pathways are used in pyrolysis to decompose these components. The depolymerization of cellulose and hemicellulose produces levoglucosan (1,6-anhydro-D-glucose), low-molecular-weight carbonyls, and furfural derivatives from pentoses. Pyrolysis of lignin produces many phenolic compounds, such as guaiacol (2-methoxyphenol), syringol (2,6-dimethoxyphenol), alkylphenols, and other substituted single-ring aromatics [17,18]. The release of these monomeric phenolics (often referred to as primary tar) typically takes place before extensive secondary reactions. During secondary reactions (cracking, oxygenation, polymerization), primary products are converted into smaller gases or larger aromatic structures as the temperature rises. High temperatures form PAHs, which are multi-ring aromatics (e.g., naphthalene, pyrene) that are toxic and tend to remain as tar or charcoal [19].

One of the most interesting biomass sources is wheat straw, which is one of the main cereals used for food production. One of the largest areas for growing this grain in the EU is the Podlaskie region in Poland. Typical agricultural conditions across Central and Eastern Europe can be found in this region due to its temperate continental climate, fertile soils, and well-established cereal farming practices. The area is one of Poland’s biggest cereal-producing areas, and wheat is one of the most commonly grown crops because it is adaptable to the local agro-climatic conditions. The European Union selected wheat as the biomass source since it is the most widely cultivated cereal crop, accounting for about 50% of total cereal production in 2023, [20,21,22]. Chemical components of wheat straw, approximately 35% of the cellulose, 25% hemicellulose, and 15% lignin, with minor amounts of proteins and ash. These values are consistent with those reported for cereal residues. Biomass was not chemically pretreated to preserve its native structure. Stored at room temperature, the prepared feedstock was kept in sealed, moisture-resistant containers [23,24,25].

The use of pyrolytic analysis coupled with a mass detector (PY/GC-MS) enables the modeling of the thermal decomposition of lignocellulosic biomass, including straw, at various process temperatures in an oxygen-free atmosphere [26,27,28]. The mass detector enables precise, often unambiguous identification of the analyzed chemical compounds based on the recording of the number of ions and their mass, creating a unique mass spectrum that acts as a “fingerprint” for each detected compound [29,30]. Unlike the FID detector, the MS detector enables precise qualitative detection of compounds. This allows for qualitative identification of compounds formed during pyrolysis. In addition, the use of this detector makes it possible to determine the relative content of the identified compounds [31,32,33]. The combination of pyrolysis with gas chromatography equipped with a mass detector (PY/GC-MS) enables the identification of decomposition products of macromolecular polymer compounds without the need for calibration to determine the identified compounds and their content in the analyzed sample. This method uses universal calibration built into the device, performed each time after column replacement, servicing, or in accordance with laboratory procedure to check the condition of the chromatograph and chromatographic column.

The distribution of biomass products is a critical transition point highlighted in the studies on biomass pyrolysis [34,35]. During wood pyrolysis, phenolic monomers from lignin dominate between 500 and 600 °C, while PAHs emerge after 700 °C as tars aromatize. Agricultural straw may follow similar trends, but its higher ash and lower lignin content may contribute to its volatile chemistry. The present study comprehensively analyzes straw pyrolysis volatiles between 350 and 1050 °C. Using pyrolysis gas chromatography–mass spectrometry (Py/GC-MS), the most significant compounds at each temperature were identified and subdivided them into chemical families (furans, phenolics, acids, esters, PAHs, etc.). The one-way ANOVA [36] was conducted for three defined temperature regimes—low (<550 °C), medium (550–850 °C), and high (>850 °C)—to assess the effect of temperature on the distribution of pyrolysis products. This statistical approach revealed significant changes in the composition of compound classes across temperature regimes, particularly for phenolics and PAHs. The study aims to understand the stepwise devolatilization mechanism of straw. The purpose of determining the modification of pyrolysis conditions is to achieve desired results using the profiles of the volatile products generated from depolymerization to aromatization. Biofuel processes should maximize liquid yield while minimizing heavy tar and PAH formation; conversely, deliberate high-temperature treatment could produce PAH-rich charcoal for specific applications.

## 2. Materials and Methods

### 2.1. Feedstock Preparation

This study used wheat straw collected from an agricultural field located in the Podlaskie Voivodeship in northeastern Poland, specifically within the Supraśl commune (53.2006° N, 23.3491° E). The straw was air-dried under ambient conditions after it was collected to reduce its moisture content. For improved heat transfer and uniformity, the biomass was mechanically milled and sieved to achieve particles smaller than 1 mm in diameter.

### 2.2. Pyrolysis Procedure

In the pyrolysis experiments, a micro-furnace pyrolyzer (Model: Frontier Labs, Koriyama, Japan, EGA/PY-3030D) was directly connected to a gas chromatograph-mass spectrometer (GC–MS; Shimadzu GC-MS-QP2010, Kyoto, Japan), enabling real-time analysis of volatile degradation products [35,37,38]. Each experimental run involved weighing and placing approximately 0.5 mg of air-dried, milled wheat straw into a deactivated quartz sample cup. To maintain an inert atmosphere and prevent secondary reactions such as oxidation or combustion, the micro-furnace was preheated to the target temperature before pyrolysis, per fast pyrolysis protocols. A continuous flow of ultra-high-purity helium gas (99%) was used to purge the system at a controlled rate, providing a stable carrier gas environment throughout the process [25]. The sample was introduced into the furnace using a drop-in mechanism, allowing immediate contact with the hot zone and facilitating rapid thermal decomposition. Pyrolysis was conducted at temperatures ranging from 350 °C to 1050 °C, in 100 °C increments; the pyrolysis process was a single-stage process. Each temperature condition was performed in triplicate to ensure statistical robustness and reproducibility. Based on prior studies, a residence time of approximately 20 seconds in the pyrolysis zone was considered sufficient for complete devolatilization of the sample [36].

Upon release, the helium stream transported volatile products directly to the split GC injection port. Compounds were separated using a fused silica capillary column (0.25 mm internal diameter) coated with 5% phenyl and 95% dimethylpolysiloxane stationary phase [6]. The column chemistry is well-suited for analyzing complex mixtures of C_1_–C_30_ organic compounds, characteristic of biomass pyrolysis vapors [39]. To facilitate the elution of high-boiling compounds, the GC oven temperature was programmed to ramp from 40 °C (with a 2 min initial hold) to 300 °C at a rate of 5 °C/min, followed by a 10 min final hold. The mass spectrometer operates in electron ionization (EI) mode, scanning a mass-to-charge (m/z) range of 15–500, thereby capturing the full range of condensable organics produced during pyrolysis. It should be noted that the samples used are small, so they cannot represent mass pyrolysis.

The analytical setup enabled semi-quantitative identification and characterization of condensable volatile products [17]. The results of this experiment may be used to observe change and trends in product distribution across temperatures, but not to determine precise compositions of pyrolysis product mixtures. Due to the lack of calibration with chemical standards, the data cannot be used to determine the absolute compositions of the pyrolysis mixtures. Gas chromatography detectors (e.g., FID, TCD) measure specific properties of compounds, such as ionization in a flame or thermal conductivity, rather than their absolute mass. Therefore, like liquid chromatography, a comprehensive calibration with chemical standards is essential for precise quantitative analysis and the determination of absolute compound concentrations. Consequently, our results enable reliable trend identification, but do not provide absolute quantification. Semi-quantitative results are suitable only for identifying trends in product formation that are dependent upon temperature.

### 2.3. Identification and Quantification

The PY/GC–MS analyses were used to identify volatile pyrolysis products and to perform semi-quantitative evaluations. Prominent chromatographic peaks were selected and matched against the NIST11 mass spectral library. Compound identification was considered reliable when the retention time and spectral match score corresponded with values previously reported for lignocellulosic biomass pyrolysis products [35,40]. Based on GC–MS results, the detected compounds were systematically grouped into prominent chemical families according to their functional groups and known pyrolysis pathways. Furans, like furfural, are typical products of hemicellulose and pentose thermal degradation. Phenolic compounds, including guaiacol, syringol, and catechol, originated from lignin depolymerization. Aldehydes and ketones were detected due to cleavage reactions in carbohydrate backbones, while carboxylic acids (e.g., formic and acetic acid) arose from oxidation and molecular rearrangements. Esters were presumed to form through the condensation of alcohols and acids. Increasing temperature, deoxygenation, and aromatization reactions led to the formation of aromatic hydrocarbons, particularly unsubstituted benzene derivatives. The presence of polycyclic aromatic hydrocarbons (PAHs)—multi-ring aromatic structures—confirmed the occurrence of advanced secondary reactions. Minor components also included aliphatic hydrocarbons and nitrogen-containing species, most likely from proteins or other non-carbohydrate biomass fractions [41,42].

The peak areas of identified compounds were integrated using GC–MS software, enabling semi-quantitative comparison. Normalized peak areas were calculated by dividing the location of each identified peak in a given total ion chromatogram (TIC) by the total integrated peak area. This method allowed for comparing relative abundances of compounds across different pyrolysis temperatures, although it did not involve internal standards, as is typical for temperature-resolved pyrolysis analysis [34,43]. To emphasize the relative nature of the presented data, consistent with the fundamental principles of GC quantification, we relied on normalized peak areas rather than performing a complete calibration with external standards for absolute quantification. The analytical window was limited to compounds in the C_1_–C_30_ range. To detect non-condensable gases, such as CO, CO_2_, H_2_, and CH_4_, alternative techniques, such as thermal conductivity detectors (TCDs) or gas analyzers, would be required [44]. The analytical framework offered insights into the evolving chemistry of pyrolysis vapors, facilitating the evaluation of lignin depolymerization, carbohydrate dehydration, and secondary tar formation and cracking. The observed trends supported the classic three-stage pyrolysis model for lignocellulosic biomass, distinguishing phenolic-rich tars formed at lower temperatures from PAH-rich tars generated under high-temperature conditions [45,46,47].

### 2.4. Statistical Analysis

The experimental data were divided into three defined thermal regimes to evaluate the impact of temperature on the distribution of pyrolysis products. The lowest temperature was in the range 350 °C, 450 °C and >550 °C, intermediate between 550 and 850 °C including 550 °C, 650 °C, 750 °C, and highest < 850 °C, in range 850 °C; 950 °C and 1050 °C. For each temperature point, the relative abundance of major compounds—including phenolics, polycyclic aromatic hydrocarbons (PAHs), and other volatiles—was calculated as a percentage of the total identified peak area [40].

To determine if there are significant differences among the three temperatures, a one-way analysis of variance was applied to each temperature run, treating each temperature as an independent experiment. Statistically significant differences were detected (*p* = 0.05), and post hoc comparisons were conducted to support the interpretation of temperature-dependent trends. The qualitative trends in the presence and evolution of individual compounds at different temperature levels were also examined to support the interpretation of chemical transformation mechanisms. Statistical analyses and visualizations were conducted using Statistica 13 (Statsoft, Warsaw, Poland) to ensure a consistent and robust assessment of temperature-dependent variations in the composition of pyrolysis products.

The statistical analysis was applied exclusively to chemically meaningful response variables derived from the GC–MS data. The pyrolysis temperature was evaluated using normalized peak areas and relative contributions of major compound classes. No statistical tests were conducted on retention time since it was only used for chromatographic separation and compound identification.

## 3. Results

### 3.1. Thermal Characterization of Chemical Compounds in Willow

#### 3.1.1. The Volatile Profile of Straw Pyrolysis at 350 °C

**Figure 1:**
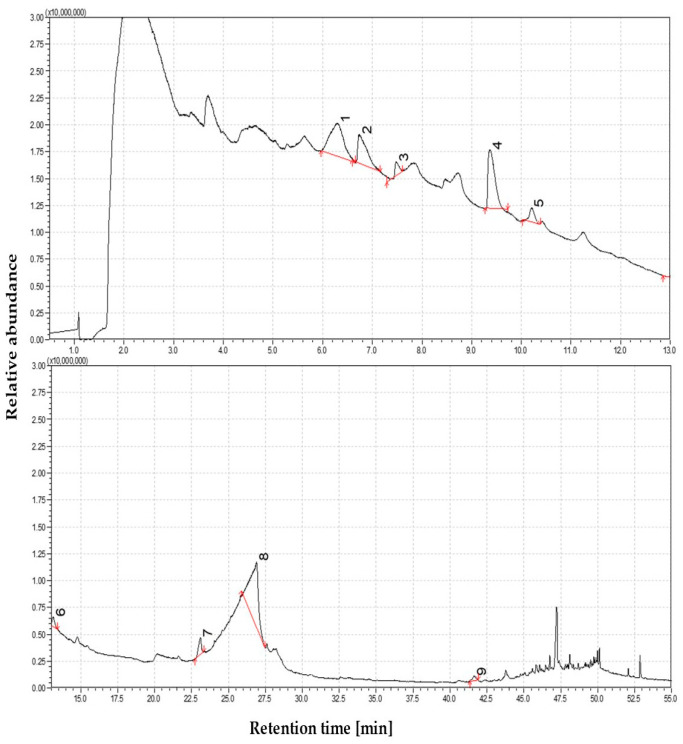
The dominance of oxygen-rich species at 350 °C—Identified compounds 1—5-Hydroxymethylfurfural; 2—4-Hydroxy-3-methylacetophenone; 3—3Allyl-6-methoxyphenol, 4—trans-Isoeugenol, 5—Phenol, 2-methoxy-4-propyl-; 6—Propan-2-one, 1-(4-isopropoxy-3-methoxyphenyl)-; 7—2-Propenal, 3-(4-hydroxy-3-methoxyphenyl)-; 8—β-D-Glucopyranose, 1,6-anhydro-; 9—3-(3-Hydroxy-4-methoxyphenyl)-l-alanine.

Pyrolysis of straw in the low-temperature regime produced a volatile mixture rich in oxygenated compounds, primarily consisting of monomeric products derived from the early-stage thermal degradation of polysaccharides and initial cleavage of lignin structures. At this stage, the total ion chromatogram (TIC) revealed relatively few distinct peaks, indicating limited volatilization and the predominance of thermally labile components [48]. Among the detected compounds, 5-hydroxymethylfurfural (5-HMF)—a furanic compound formed from the degradation of hexose sugars—was one of the most abundant. An even greater contribution came from levoglucosan (1,6-anhydro-β-D-glucopyranose), a key cellulose pyrolysis marker, accounting for more than half of the identified volatiles. The high abundance of levoglucosan and related anhydrosugars, such as 1,5-anhydroglucitol, suggests cellulose undergoes partial depolymerization under mild thermal conditions, yielding high-molecular-weight oxygen-rich tar species without extensive fragmentation. As illustrated in the corresponding pie chart, this early-stage product profile reflects the dominance of oxygenated species typical for low-temperature pyrolysis conditions. The total ion chromatogram (TIC) of volatile products from straw pyrolysis at 350 °C is presented in Figure 1.

Despite the relatively low thermal conditions, the number of phenolic compounds originating from lignin degradation was identified. Guaiacol and its alkylated derivatives—including 4-hydroxy-3-methylacetophenone, 3-allyl-6-methoxyphenol, and isoeugenol (trans-4-propenyl-2-methoxyphenol)—were the most prominent peaks. These compounds suggest cleavage of ether bonds, particularly within guaiacyl lignin substructures. The monoaromatic phenolics observed at this stage were exclusively oxygenated lignin-derived products, bearing functional substituents such as hydroxyl and methoxy groups. No polycyclic aromatic hydrocarbons (PAHs) or other multi-ring aromatics were detected, confirming the absence of condensation products typical of higher-temperature regimes. In addition, several minor constituents were observed, including aliphatic hydrocarbons such as 1-nonene, and a nitrogen-containing compound—3-(3-hydroxy-4-methoxyphenyl)-L-alanine, which likely formed via thermolytic decomposition of proteinaceous biomass components. The high proportion of reactive, oxygen-rich volatiles at this low temperature indicates a product spectrum characteristic of primary pyrolysis tars. These are typically composed of functionalized intermediates resulting from the initial fragmentation of biomass macromolecules, and they lack the condensed aromatics associated with secondary tar formation.

#### 3.1.2. The Volatile Profile of Straw Pyrolysis at 450 °C

**Figure 2:**
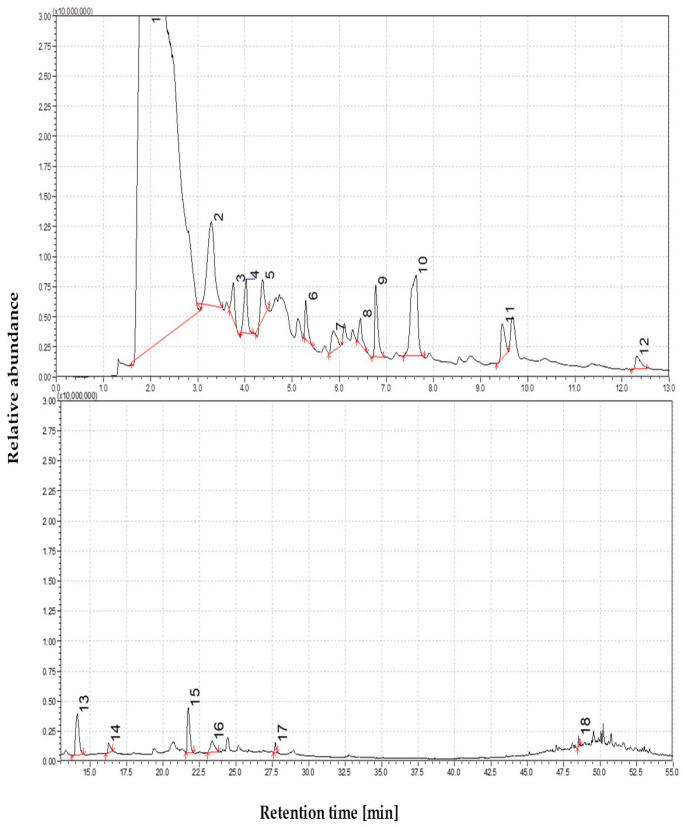
The dominance of oxygen-rich species at 450 °C; Identified compounds 1—1,4-Butanediol; 2—Formic acid, heptyl ester; 3—3-Cyclobutene-1,2-dione, 3,4-dihydroxy-; 4—2-Cyclopenten-1-one, 2-hydroxy-3-methyl-, 5—Phenol, 2-methoxy-; 6—Creosol; 7—Catechol; 8—1,2-Benzenediol, 3-methoxy-; 9—2-Methoxy-4-vinylphenol; 10—Phenol, 2,6-dimethoxy-; 11—Phenol, 2-methoxy-4-(1-propenyl)-, (Z)-; 12—Benzene, 1,2,3-trimethoxy-5-methyl-; 13—3′,5′-Dimethoxyacetophenone; 14—Phenol, 2,6-dimethoxy-4-(2-propenyl)-; 15—Phenol, 2,6-dimethoxy-4-(2-propenyl)-; 16—Ethanone, 1-(4-hydroxy-3,5-dimethoxyphenyl)-; 17—n-Hexadecanoic acid; 18—Mandelic acid, 3,4-dimethoxy-, methyl ester.

The devolatilization of straw at moderate thermal conditions proceeds more extensively than at lower temperatures, resulting in a broader spectrum of pyrolysis products. The total ion chromatogram (TIC) recorded at this stage revealed an increased number and intensity of peaks, indicating a higher release of volatile compounds. The enhanced chemical complexity was reflected in the dominance of lignin-derived phenolic compounds within the chromatographic profile. Regarding relative peak area, creosol (4-methylguaiacol) accounted for approximately 21%, suggesting cleavage of methoxy groups from guaiacyl and syringyl lignin subunits during depolymerization. Resorcinol (1,3-dihydroxybenzene) was present at 16.3%, likely formed via demethylation of guaiacol. The guaiacol (2-methoxyphenol) peak was also detected, albeit at a lower abundance (1.1%). Other resolved alkylated derivatives, such as 4-vinylguaiacol (10.5%) and vanillin (8%), further support the lignin matrix’s progressive depolymerization and side-chain modification. The products included phenol and 2-methoxy-4-(2-propenyl) acetate (~4.5%), an esterified eugenol derivative. The presence of this compound suggests that acetylation reactions may occur either as part of the pyrolysis process or due to residual extractives present in the raw straw. The results indicate that bond cleavage within lignin intensifies progressively across low to moderate temperatures, promoting the diversification of monoaromatic phenolics. The TIC profile and compound annotations for this temperature, which emphasize key compositional shifts relative to lower pyrolysis conditions, are shown in Figure 2.

The pyrolysis of straw at moderate temperatures resulted in a marked increase in the diversity and intensity of chromatographic peaks compared to lower thermal conditions. Although cellulose and hemicellulose degradation products were still present, their relative abundance was notably reduced compared to those observed at lower temperatures. Levoglucosan, the primary marker of carbohydrate pyrolysis, declined to approximately 3% of the total volatile area, suggesting that it had either already been depleted through earlier depolymerization or had undergone secondary decomposition reactions. Continued hemicellulose degradation was reflected in detecting minor furan derivatives, such as 5-methylfurfural (1–2%). The detection of 1-nonanol (~4%), a long-chain aliphatic alcohol, likely originating from cuticular waxes or suberin-like lipids, further supports the volatilization of non-structural straw constituents under these conditions. The total ion chromatogram (TIC) at this temperature showed an apparent increase in the number of peaks and overall signal intensity, compared to the lower-level profile. This shift reflects a transition from simple carbohydrate-derived volatiles to a more chemically complex tar matrix, increasingly enriched in lignin-derived aromatic compounds.

The majority of compounds detected at this stage were monoaromatic phenolics, including guaiacols, cresols, and catechols, which are formed through the cleavage of β-O-4 ether linkages and subsequent side-chain transformations within the guaiacyl and syringyl subunits of lignin. Notably, no polycyclic aromatic hydrocarbons (PAHs) were identified, confirming that 450 °C remains below the threshold temperature required for secondary condensation reactions leading to fused-ring structures. Among the most abundant products were four well-known phenolic pyrolysis markers: creosol (21%), resorcinol (16%), 4-vinylguaiacol (10%), and vanillin (8%), all of which reflect progressive lignin depolymerization. Quantitative analysis indicated that phenolics accounted for approximately 50% of the total volatile composition, sugar- and furan-derived compounds represented around 35%, and aliphatic and nitrogen-containing species comprised the remaining 15%. As the pyrolysis temperature increases, the relative contribution of lignin-derived tars becomes more dominant, and pyrolysis at this stage yields a vapor phase rich in monoaromatic phenolics, typical of low-temperature, primary tar formation.

#### 3.1.3. The Volatile Profile of Straw Pyrolysis at 550 °C

**Figure 3:**
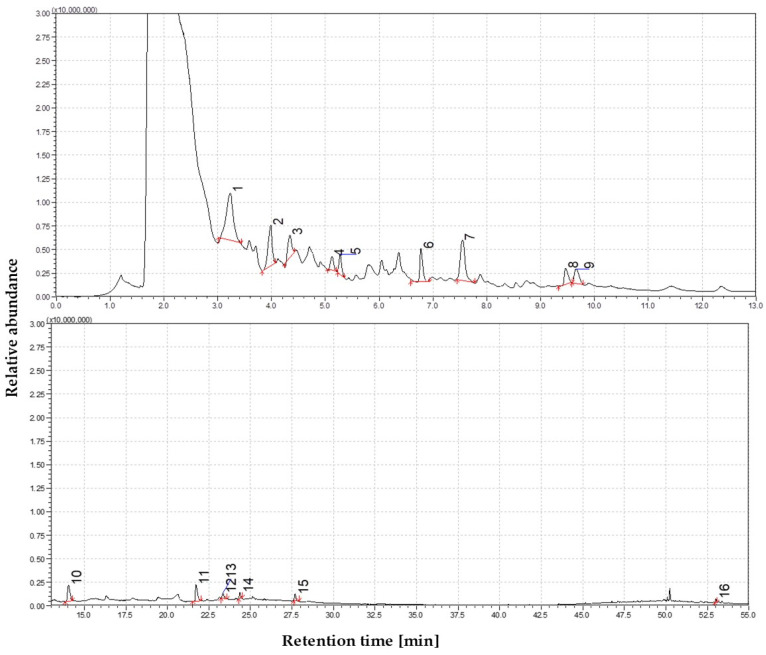
The dominance of oxygen-rich species at 550 °C; Identified compounds 1—Cyclohexanone; 2—2-Cyclopenten-1-one, 2-hydroxy-3-methyl-; 3—Phenol, 2-methoxy-; 4—1,4-Benzenediol, 2,5-dimethyl-; 5—Creosol; 6—4-Hydroxy-2-methylacetophenone; 7—Phenol, 2,6-dimethoxy-; 8—Phenol, 2-methoxy-4-(1-propenyl)-, (Z)-; 9—Phenol, 4-methoxy-3-(methoxymethyl)-; 10—3′,5′-Dimethoxyacetophenone; 11—Phenol, 2,6-dimethoxy-4-(2-propenyl)-; 12—Ethanone, 1-(4-hydroxy-3,5-dimethoxyphenyl)-; 13—Ethanone, 1-(4-hydroxy-3,5-dimethoxyphenyl)-; 14—2-Pentanone, 1-(2,4,6-trihydroxyphenyl); 15—n-Hexadecanoic acid; 16—Stigmasta-3,5-dien-7-one.

The distribution of volatile products at 550 °C reflects the influence of secondary cracking and molecular rearrangement reactions. Compared to 450 °C, phenolic compounds dominate, but their composition shifts noticeably. A marked increase in phenol (C_6_H_5_OH) concentration is observed, likely due to thermal demethoxylation of guaiacol- and syringol-derived intermediates. The removal of methoxy groups and C_3_ side chains further simplifies lignin-derived structures, resulting in the formation of o-cresol (2-methylphenol) and catechol (1,2-benzenediol) as major constituents. Detecting methylated catechols, such as 4-methylcatechol, indicates a progressive breakdown of more complex guaiacyl units. These observations suggest that substituted lignin monomers increasingly become simpler monoaromatic compounds, typically bearing only hydroxyl or methyl substituents. Compared to lower temperatures, the volatile fraction at 550 °C is more chemically functionalized, reflecting the lignin matrix’s advanced depolymerization and thermal degradation. This behavior is consistent with established lignin pyrolysis pathways, where the prevalence of single-carbon cleavage reactions promotes aromatic ring modifications and subsequent secondary transformations at elevated temperatures. The combined effects of these mechanisms signal a chemical transition toward the generation of thermally evolved tars, characterized by structurally simplified but functionally enriched phenolic compounds. The distribution and relative intensities of these compounds are illustrated in Figure 3.

The contribution of carbohydrate-derived products further decreases at this temperature. Levoglucosan is almost absent (~1%), and furan derivatives, such as furfural-related compounds, are detected only in trace amounts (~2%). In contrast to these diminishing products, smaller carbonyl compounds emerge, including 1-cyclopentanedione, 3-methyl-2-furaldehyde, and 5-ethyl-2-furaldehyde, likely originating from secondary cracking of sugar-derived intermediates. Nonanoic acid (~2%) is also detected, possibly formed through the oxidation of aliphatic fragments or thermolysis of fatty acids naturally present in the biomass. While polycyclic aromatic hydrocarbons (PAHs) remain undetected at 550 °C, the presence of nitrogen-containing species such as 2(1H)-pyridinone and 1-cyclohexyl-3,4,5,6-tetramethylpyridine suggests that ammonia release or proteinaceous nitrogen plays an early role in tar chemistry. It is also possible that aniline derivatives are present but masked under broad phenolic signals. The volatile mixture at 550 °C still corresponds to primary tar, although it already exhibits signs of secondary alteration. As the presence of carbonyl-oxygenated hydrocarbons (COH) diminishes, phenolics dominate the volatile fraction (~65%), with increasing contributions from smaller carbonyls and light acids (5–10%). Based on these findings, 550 °C remains below the condensation threshold required for fused-ring aromatic formation. One-way ANOVA analysis showed that sugar-derived compounds and PAHs were significantly more prevalent at lower temperatures (350 and 450 °C) compared to higher ranges (550–850 °C) (*p* = 0.01). However, within this broad range, 550 °C still belongs to the low-temperature end, consistent with the non-detection of PAHs.

#### 3.1.4. The Volatile Profile of Straw Pyrolysis at 650 °C

**Figure 4:**
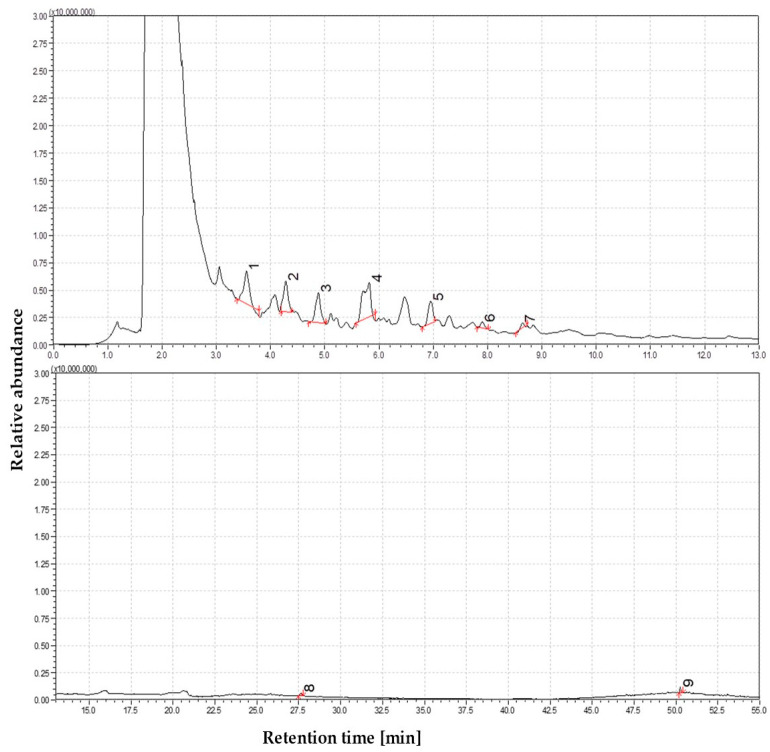
Total ion chromatogram (TIC) of volatile products from straw pyrolysis at 650 °C. Identified compounds 1—Carbamic acid, phenyl ester; 2—Phenol, 3-methyl-; 3—Phenol, 2,6-dimethyl-; 4—Catechol; 5—1,2-Benzenediol, 4-methyl-; 6—1,4-Benzenediol, 2,6-dimethyl-; 7—4-Ethylcatechol; 8—n-Hexadecanoic acid(Z)-; 9—Acetic acid, 3-hydroxy-6-isopropenyl-4,8a-dimethyl-1,2,3,5,6,7,8,8a-octahydronaphthalen-2-yl ester.

The effects of secondary cracking, demethoxylation, and initial aromatization become increasingly evident at 650 °C. Although phenolic monomers continue to dominate the volatile product spectrum, they appear chemically simpler and more deoxygenated compared to those formed at lower temperatures. A further increase in phenol concentration is observed, along with elevated levels of o-cresol, m-cresol, dimethylphenols (e.g., 2,4-dimethylphenol), and ethylphenols, indicating ongoing degradation of alkylated and methoxylated lignin derivatives. The chromatographic profile comprises alkyl-substituted phenols, suggesting progressive side-chain scission and substitution on the aromatic rings. Catechols and methylcatechols, prominent at earlier stages, undergo dehydration and decarbonylation, forming simpler monoaromatic structures. Protocatechuic acid, known to decarboxylate under pyrolytic conditions, may contribute to these transformations. Notably, straw pyrolysis at this temperature yields the first detection of two-ring aromatic structures, indicating the onset of polycyclic formation, potentially via cyclization reactions or Diels–Alder-type mechanisms. A related compound, 2,5-dimethylbenzoic acid (~1–2%), was also detected, probably originating from the oxidation of methylphenols. The detection of oxygenated polyaromatic compounds further supports the occurrence of early-stage polycyclic evolution. The complete distribution of compounds and the corresponding peak intensity trends at this temperature are shown in Figure 4.

Nitrogen-containing components and trace components began to emerge simultaneously. Two of these peaks, which may represent partial isomers or duplicate identifications, contained phenyl carbamate, an aromatic nitrogen compound. The formation of carbamate structures suggests phenols or isocyanate-like intermediates react with nitrogen from straw proteins or amides. In addition to p-tert-octylphenol (1%), another compound was detected. Although not associated with biomass, its presence may indicate trace contamination by antioxidants or plastic. The detection threshold for non-substituted PAHs like naphthalene was not reached, and co-eluting phenolics, which obscured them, and other compounds like biphenyl were marginally present at about 0.5%. Levoglucosan remained traces of life despite the rising room temperature, indicating that small fragments of carbohydrates survive even after the onset of melting. Approximately 75% of the volatiles identified were phenolics, 10% acids and esters, and only a few aromatic nitrogen compounds. In biomass pyrolysis, PAHs rarely form at 650 °C because the literature often cites 600 °C as the lower limit for their formation. As in our experiments, secondary condensation in the pyrolyzer could be limited by the short vapor residence time of the micropyrolysis products.

#### 3.1.5. The Volatile Profile of Straw Pyrolysis at 750 °C

**Figure 5:**
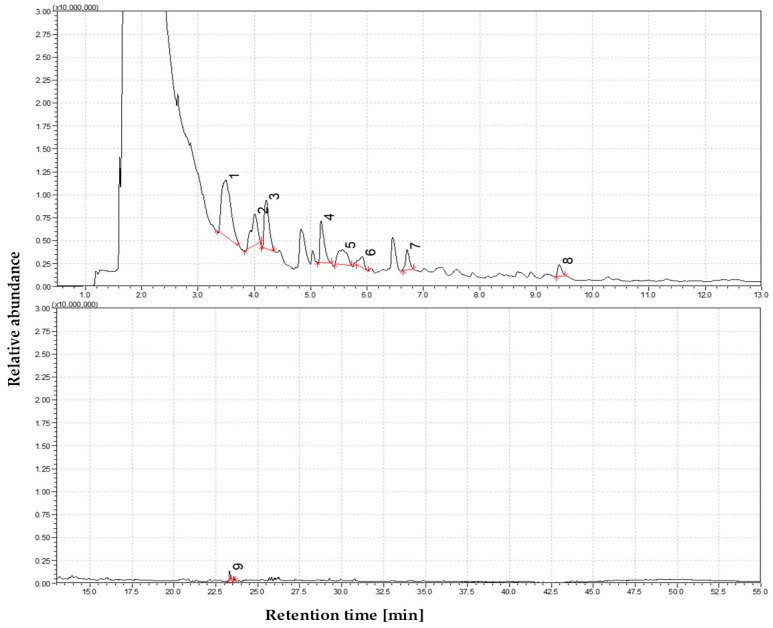
Total ion chromatogram (TIC) of volatile products from straw pyrolysis at 750 °C. Identified compounds 1—Phenol; 2—Indene; 3—Phenol, 3-methyl-; 4—Naphthalene; 5—Benzofuran, 2,3-dihydro-; 6—Phenol, 2-ethoxy-; 7—Naphthalene, 2-methyl-; 8—Acenaphthylene; 9—Anthracene.

The pyrolysis of straw crosses a significant chemical threshold at 750 °C, marked by a noticeable increase in polycyclic aromatic hydrocarbons (PAHs) and a rapid decline in oxygenated species. Naphthalene (C_10_H_8_) and its alkylated derivatives exhibit distinct peaks at this temperature for the first time. Approximately 10–15% of the total ion chromatogram (TIC) area is occupied by two closely eluting peaks, likely corresponding to indene or its isomeric forms. Several three-ring PAHs, including fluorene and anthracene/phenanthrene, are detected in trace amounts, while four-ring PAHs, such as pyrene and benz[a]anthracene, remain absent. Under high-temperature, radical-rich conditions, smaller aromatic species—such as phenol, indene, and benzyl radicals—undergo dehydrogenation, condensation, and ring fusion, promoting the formation of fused-ring structures. These transformations provide evidence of a transition toward tertiary tar formation regimes, characterized by increasing aromaticity and structural complexity. The range of compounds generated at this stage and their distribution across retention times are illustrated in Figure 5.

The monoaromatic phenolics, however, remain a significant component of the volatile output despite the shift toward polyaromatic species. In addition to phenols, alkylphenols—including cresols and ethylphenols—contribute approximately 10% of the total volatile composition. Catechol is also detected at a reduced intensity (~1%), indicating that some phenolic substructures can survive even under intense pyrolytic conditions. Interestingly, this temperature is atypical for forming 5-hydroxymethylfurfural (HMF), suggesting either rapid quenching of intermediates or incomplete degradation of carbohydrate residues. Chromatographic analysis also reveals a prominent peak for phenylacetylene (~14%), whose presence likely results from dehydrogenation or recombination of styrene, phenyl, and acetylene radicals, indicative of an unsaturated and highly reactive environment. Minor components, such as phenyl carbamate and p-tert-octylphenol, may form through interactions between phenolic compounds and nitrogen sources (e.g., ammonia or protein-derived intermediates) or may stem from external contamination. The chemical profile at 750 °C reflects an ongoing transformation toward more condensed structures. The proportion of PAHs (compounds with two or more rings) increases substantially from negligible levels at 650 °C to approximately 35–40% of the identified products.

In contrast, phenolics, which dominated at lower temperatures, decrease to about 50%, continuing their downward trend. The remaining 10–15% of the volatile fraction comprises non-oxygenated monoaromatics, small acids/esters, and nitrogen-containing species. Levoglucosan is still detectable at a low level (~0.8%), suggesting that some carbohydrate residues resist complete cracking in localized microenvironments. Across the 550–850 °C range, phenolics constitute around 60%, PAHs approximately 18%, and other compounds about 22% of the volatile output; however, specifically at 750 °C, phenolics and polyaromatics account for nearly equal proportions. These shifts indicate that straw pyrolysis at 750 °C produces tars with higher carbon content, lower oxygen content, and reduced volatility, signaling the transition toward more thermally evolved tar structures.

#### 3.1.6. The Volatile Profile of Straw Pyrolysis at 850 °C

**Figure 6:**
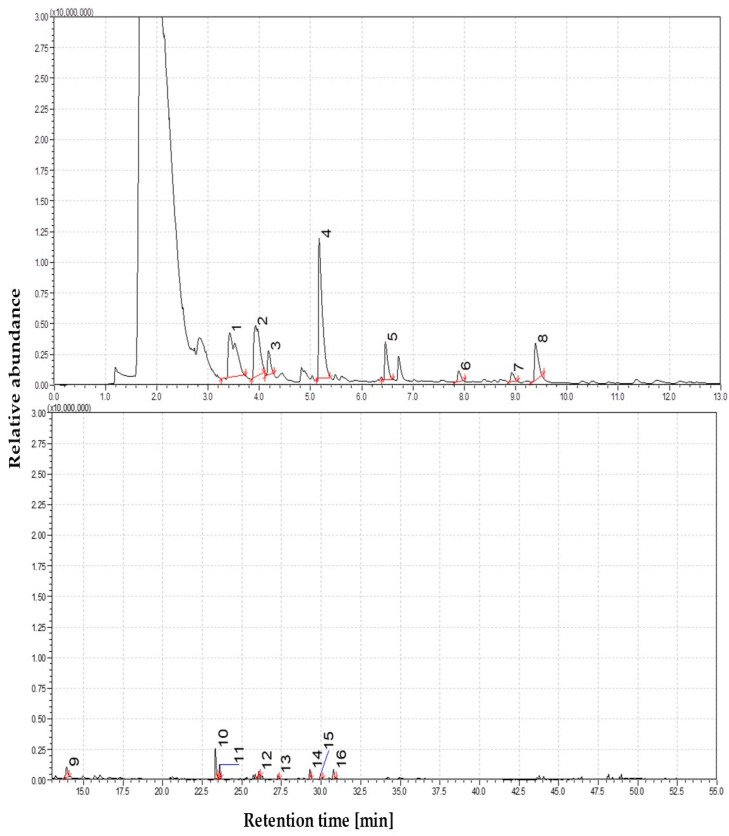
Total ion chromatogram (TIC) of volatile products from straw pyrolysis at 850 °C. Identified compounds 1—Phenol; 2–Indene; 3—Phenol, 3-methyl-; 4—1Naphthalene; 5—Naphthalene, 1-methyl-; 6, 7—Biphenyl; 8—Biphenylene; 9—Fluorene; 10, 11—Anthracene; 12—4H-Cyclopenta[def]phenanthrene)-; 13—Naphthalene, 2-phenyl-; 14—Pyrene; 15—Fluoranthene; 16—Pyrene.

The pyrolysis of straw progresses into a tertiary tar formation regime at 850 °C, where polycyclic aromatic hydrocarbons (PAHs) begin to dominate, and oxygenated species are further diminished. Even though the overall distribution of volatiles remains qualitatively similar at 750 °C, major shifts are evident. Phenol remains the most abundant compound (24% of total area), reflecting continued volatilization of lignin-derived compounds. Other oxygenates, such as cresols (13%) and catechols (2–3%), are declining. About 35–40% of condensable products are PAHs, including naphthalene at 30%, possibly due to the cracking of large rings or condensation from lighter aromatics. The residual oxygenated (0.6%), fluorene, pyrene, and benz[a]anthracene also appear as dibenzofuran molecules in trace amounts. The total ion chromatogram (TIC) of volatile products from straw pyrolysis at 850 °C is presented in Figure 6.

The pyrolysis of straw at 850 °C continues the trends observed at 750 °C, while highlighting essential nuances. Despite the increasing prevalence of polycyclic aromatic hydrocarbons (PAHs), not all volatile species are fully aromatized. Phenylacetylene (~14%) remains persistent, suggesting that phenyl radicals can stabilize through reactions with unsaturated fragments rather than directly progressing toward PAH formation. A small but consistent signal of 5-hydroxymethylfurfural (HMF, ~1.5%) is also detected, likely originating from residual carbohydrate structures or resulting from rapid local quenching. These findings suggest that microenvironmental factors or kinetic limitations may hinder complete aromatization, even under elevated temperature conditions. Analysis of the volatile mixture reveals the coexistence of radical-stabilized monoaromatic compounds and persistent intermediates, pointing to a complex interplay between secondary cracking, radical recombination, and incomplete devolatilization. The vapors produced in this thermal regime are chemically complex, containing refractory PAHs and residual phenolic monomers. Process parameters, such as vapor residence time and heating rate, can be tuned to favor phenolic retention or suppress excessive PAH formation. Consequently, this temperature window is critical for optimizing the quality and yield of biomass pyrolysis products.

#### 3.1.7. The Volatile Profile of Straw Pyrolysis at 950 °C

**Figure 7:**
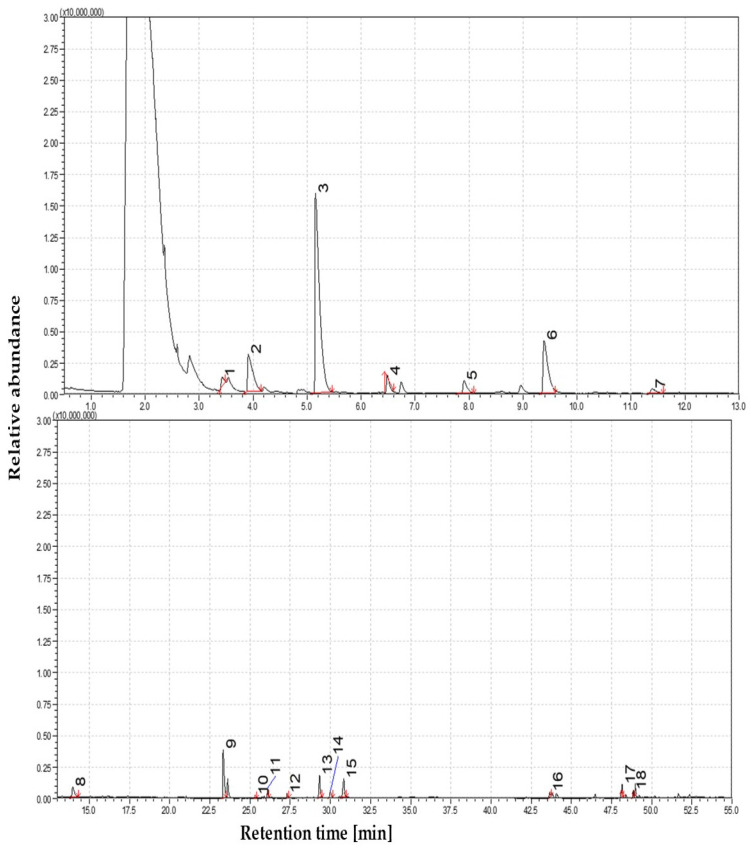
Total ion chromatogram (TIC) of volatile products from straw pyrolysis at 950 °C. Identified compounds 1—Formic acid phenyl ester; 2—Indene; 3—Naphthalene; 4—Naphthalene, 1-methyl-, 5—Biphenyl; 6—Biphenylene; 7—Dibenzofuran; 8—Fluorene; 9—Anthracene; 10—Anthracene, 9-ethenyl-; 11—4H-Cyclopenta[def]phenanthrene; 12—Naphthalene, 2-phenyl-; 13, 15—Pyrene; 14—Fluoranthene; 16, 17—Benzo[ghi]fluoranthene; 18—Benzo[j]fluoranthene.

The volatiles released during straw pyrolysis reach a highly aromatic regime at 950 °C, with virtually no oxygenated compounds remaining. According to the GC–MS chromatogram, increasing complexity and molecular weight PAHs are detected. Nearly 90% of the identified compounds are PAHs, reflecting extensive secondary condensation, cyclization, and polymerization processes. Major detected PAHs include methylene, methyl-naphthalene, indene, biphenyl, fluorene, anthracene, phenanthrene, as well as larger structures such as pyrene, benz[a]anthracene, benzo[ghi]fluoranthene, and perylene. Multiple isomeric forms of several species are observed, indicating extensive ring rearrangement and molecular reorganization. These findings suggest that the tar produced at this temperature has undergone advanced thermal maturation, consolidating into heavy aromatics and approaching the stage of soot precursors, particularly based on the appearance of four- and five-ring PAHs. At such high temperatures, gasification and pyrolysis processes drive volatiles toward fragmentation into small gas molecules or condensation into increasingly stable polycyclic aromatic structures. The Total ion chromatogram (TIC) of volatile products from straw pyrolysis at 950 °C is presented in Figure 7.

The volatile products at 950 °C contain almost no oxygenated compounds. Phenol remains detectable at approximately 3.6%, likely formed through char-mediated recombination or the continuous release of phenol from residual lignin structures, representing one of the few surviving oxygen-containing species. Other oxygenates, such as dibenzofuran and benzofuran, are present only in trace amounts. A rare exception is vinyl formate (~0.8%), a volatile ester likely produced by the interaction of formic acid and acetylene, or via radical-driven mechanisms. This suggests that even under extreme thermal conditions, small amounts of oxygen released from cellulose degradation can recombine with unsaturated fragments. Additionally, azulene (~1%), a thermal rearrangement isomer of naphthalene, is detected, indicating active high-temperature PAH isomerization processes. The volatile profile at this stage is overwhelmingly dominated by polycyclic aromatic hydrocarbons (PAHs, ~89%), with minor contributions from phenolics (~3.6%) and esters (~1%). These results confirm that secondary and tertiary tars at this temperature primarily comprise condensed aromatic structures. During this advanced stage of thermal evolution, stable, high-molecular-weight aromatics vastly outnumber lighter, oxygenated intermediates. Understanding the chemical nature of vapors at 950 °C is crucial, as tars formed under these conditions are less likely to condense into liquid phases and are more prone to contribute to soot formation or catalyst fouling in high-temperature biomass conversion processes.

#### 3.1.8. The Volatile Profile of Straw Pyrolysis at 1050 °C

The pyrolysis of straw at 1050 °C approaches the conditions typical of flame pyrolysis or entrained-flow gasification, with PAHs dominating the volatile products and minimal oxygenated species remaining. Although other volatiles, such as esters, are still detected, more than 75% of the identified tar compounds consist of PAHs, slightly lower than the proportion observed at 950 °C. Several PAHs are found in the samples, including naphthalene, fluorene, biphenyl, anthracene, pyrene, and indeno[1,2,3-cd] fluoranthene. Notably, phenanthrene and pyrene are less abundant at temperatures above 950 °C, suggesting that these compounds undergo cracking at higher temperatures. Observations also indicate an increase in 1H-phenalene (C_13_H_10_) and related C_13_H_10_ species, which are recognized intermediates in PAH degradation pathways. The volatile profile suggests that while PAHs are still generated, they are partially fragmented into smaller aromatic structures or retained within solid charcoal or soot precursors, reducing their detectability in the gas phase. One of the most unexpected results at 1050 °C is the high intensity of diethyl carbonate (DEC, ~21%), which becomes the most abundant single peak in the chromatogram.

**Figure 8:**
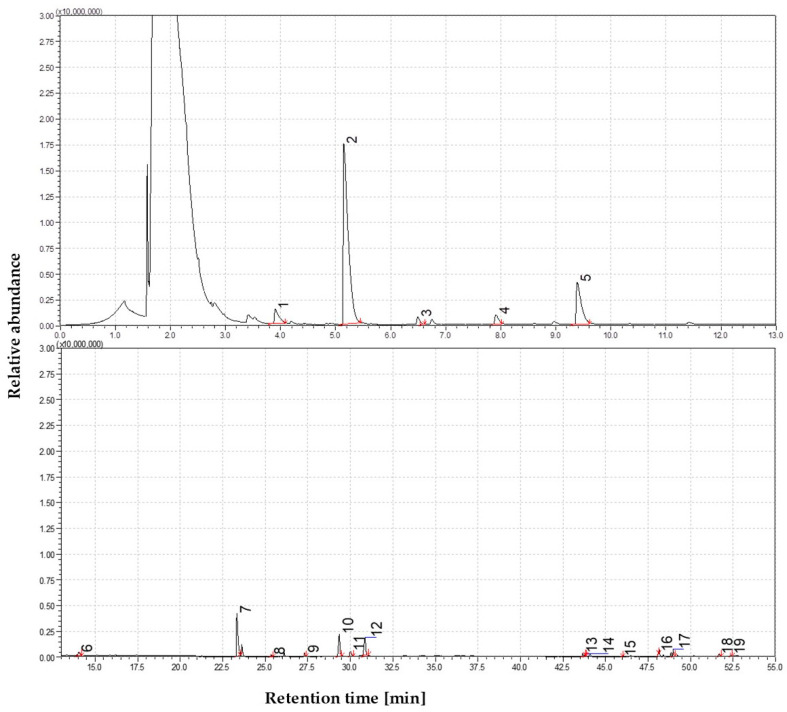
Total ion chromatogram (TIC) of volatile products from straw pyrolysis at 1050 °C. Identified compounds 1—Indene; 2—Naphthalene; 3—Naphthalene, 1-methyl-, 4—Biphenyl; 5—Biphenylene; 6—Fluorene; 7—Anthracene; 8—Anthracene, 9-ethenyl-; 9—Naphthalene, 2-phenyl-; 10, 12—Pyrene; 11—Fluoranthene; 13—Benzo[ghi]fluoranthene; 14—Benz[a]anthracene, 15—Benzo[c]phenanthrene, 1-methyl-; 16, 17—Benzo[j]fluoranthene; 18—Indeno[1,2,3-cd]pyrene; 19—Benzo[ghi]perylene.

In the absence of moisture, DEC, a volatile ester of carbonic acid, exhibits unusual behavior under pyrolytic conditions. A plausible formation mechanism involves the reaction of ethanol, produced during the thermal decomposition of cellulose and hemicellulose, with carbon dioxide and ethyl radicals. Under oxygen-deficient, high-temperature conditions, radical recombination between ethoxy fragments and CO_2_ may form diethyl carbonate through an in situ esterification process. This mechanism is consistent with radical chemistry observed in pyrolysis systems and explains the unexpected abundance of DEC at this temperature. Detecting vinyl formate (~3%) further indicates that radical recombination can produce volatile esters even under low-oxygen, high-temperature conditions. Although oxygenates remain minor relative to the aromatic load, they are significant enough to reduce the relative proportion of PAHs. Only a trace amount of phenol (~3.9%) is detected, likely resulting from secondary decomposition or radical recombination processes. The GC-detectable tar yield at 1050 °C is the lowest among all temperatures studied, possibly due to extensive cracking or condensation into non-volatile soot precursors. The overall composition of compounds in the >850 °C regime is illustrated in the rightmost segment of Figure 8.

The endpoint of straw pyrolysis occurs at 1050 °C, where aromatic condensation and deoxygenation are nearly complete, although some light oxygenates may still form via radical-mediated reactions. Compared to 950 °C, no significant difference in PAH content is observed; however, a notable increase in ester compounds, primarily due to diethyl carbonate (DEC), is detected. Despite being a system-specific artifact, this observation underscores that trace compounds can significantly impact the apparent composition of tar, even during the advanced stages of pyrolysis. While such high temperatures are typically unsuitable for bio-oil production due to low liquid yields and elevated PAH concentrations, they may be advantageous for processes aimed at complete tar removal or for cracking tars into syngas during gasification or high-temperature reforming. Thermal devolatilization at 1050 °C thus results in the survival of the most stable aromatic structures and light radical species.

The thermal decomposition of wheat straw and related lignocellulosic biomass has been investigated using pyrolysis-gas chromatography/mass spectrometry (Py-GC/MS). For instance, Rouches et al. [49,50] observed that phenolic compounds dominate at mid-temperatures (500–700 °C), whereas PAHs increase significantly above 800 °C, which is in agreement with our results. A similar temperature-dependent evolution of phenolic and aromatic compounds was also reported by Liu et al. (2008) [51], confirming trends observed in this study. According to Wądrzyk et al. [52], pyrolysis temperatures increase with oxygenated volatiles evolving into polyaromatics. The range of temperatures we covered in our study (350–1050 °C) confirms the typical trends but identifies unique compounds, such as diethyl carbonate, at the highest temperature. There is general consistency across studies supporting the reliability of temperature-dependent product evolution in straw pyrolysis. By comparing our experimental findings to those in the broader field of biomass pyrolysis, we confirm both the validity of our observations and the relevance of our research findings [53].

### 3.2. Statistical Comparison of Volatile Compounds

The influence of pyrolysis temperature on volatile product formation was evaluated based on normalized GC–MS peak areas and the relative contributions of major compound classes. To facilitate interpretation, the experimental temperatures were grouped into three regimes: low (<550 °C), intermediate (550–850 °C), and high (>850 °C). The analysis focused exclusively on chemically meaningful response variables describing changes in volatile composition.

At temperatures below 550 °C, the volatile fraction was dominated by oxygenated compounds derived from the primary thermal decomposition of carbohydrates and lignin. Phenolic compounds accounted for approximately 49.6% of the identified volatiles, while sugar-derived compounds represented about 27.9%. Polycyclic aromatic hydrocarbons (PAHs) were absent or present only in trace amounts, indicating that secondary condensation reactions had not yet occurred.

In the intermediate temperature regime (550–850 °C), phenolic compounds reached their maximum relative contribution, accounting for approximately 60.0% of the volatile fraction. This temperature window corresponds to advanced lignin depolymerization and secondary modification of phenolic intermediates. In parallel, PAHs began to emerge, representing approximately 17.8% of the volatile products, reflecting the onset of aromatization and early polycondensation reactions.

At temperatures exceeding 850 °C, the volatile composition shifted markedly toward highly condensed aromatic structures. PAHs became the dominant compound class, accounting for approximately 76.4% of the identified volatiles, whereas the relative contribution of phenolic compounds declined sharply to about 6.3%. The remaining fraction consisted mainly of esters and light aromatic hydrocarbons (approximately 12.7%), which are attributed to secondary cracking and radical recombination reactions occurring under high-temperature conditions.

Statistical evaluation based on normalized peak areas confirmed that the distribution of volatile compound classes is strongly dependent on pyrolysis temperature. The observed progression from oxygenated, phenolic-rich volatiles at low temperatures to PAH-dominated profiles at high temperatures reflects the transition from primary to secondary and tertiary tar formation during straw pyrolysis.

## 4. Discussion

Based on these results, straw pyrolysis exhibits a transparent and progressive chemical transformation, primarily driven by temperature. As the temperature decreases (350–450 °C), cellulose and hemicellulose depolymerize, resulting in high concentrations of oxygenated compounds such as levoglucosan and furanoid derivatives (e.g., 5-HMF). Devolatilized species such as these are uncondensed, carbohydrate-rich tars that represent an early stage of devolatilization. A similar result is observed when carbohydrate-derived volatiles are pyrolyzed from rice straw and sawdust at 400 °C [54]. At temperatures of 550 °C or higher, lignin decomposition takes precedence. During the depolymerization of lignin, which occurs around 350 °C, other phenolic monomers such as guaiacol, cresols, and cresols are released. In woody biomass studies, functionalized phenolics dominate tar composition in this window [55,56]. According to the analysis, phenolics make up to 60% of the total volatile fraction between 550 °C and 850 °C, peaking at 650 °C. In contrast, PAHs are almost absent in this range, and residual carbohydrate markers, such as levoglucosan, are still detectable, albeit at a decreasing level.

The rate of polycondensation and dehydrogenation increases as the temperature increases beyond 650 °C. It is noted that the first PAHs—naphthalene, indene, and phenylacetylene—emerge around 750 °C, similar to the results in willow pyrolysis [57]. Compounds formed from the recombination and cyclization of phenols are known as tertiary tars. The signal intensity of phenolic compounds decreases with increasing retention times. A significant portion of the volatile profile above 850 °C is composed of PAHs, with 76.4% of the compounds identified as PAHs at or above that temperature. The phenolic compounds decline sharply to 6.3%, reflecting both their decomposition and transformation into a more thermally stable polyaromatic form. It is accompanied by the appearance of heavier PAHs (e.g., pyrene, fluoranthene, benz[a]anthracene), which are linked to environmental and health hazards due to their long-lasting persistence and toxicity [58]. Compositional changes mark a significant shift from bio-oil-like vapors to soot precursors. Controlling the pyrolysis temperature is crucial for tailoring the product distribution and minimizing the emission of toxic gases [59,60,61].

The observed compositional changes indicate that the pyrolysis temperature controls the evolution of volatile products during straw pyrolysis. Increasing thermal severity induces a gradual shift away from oxygenated, phenolic-rich mixtures toward highly condensed, PAH-dominated profiles. As a result of normalizing GC-MS peak areas, these trends reflect chemical transformation pathways rather than chromatographic parameters. Temperature plays a major role in the transition between primary, secondary, and tertiary tar formation.

The implications of these chemical and statistical insights for biomass utilization are significant. A temperature range of 450 to 550 °C is preferred for maximizing liquid yields and preserving valuable phenolics, such as guaiacol and vanillin, in bio-oil production. To improve fuel properties, these compounds must be upgraded due to their oxygenated nature. Syngas generation or biocharcoal production requires higher temperatures (>850 °C) to enhance deoxygenation, but elevated PAH levels pose a challenge. Environmental concerns are raised when toxic PAHs—such as benzo/[ghi]fluoranthene, benz/[a]anthracene, and indeno/[1,2,3-cd]pyrene—are detected. The EPA [62] and IARC [63] list these compounds as Group 1 or 2 carcinogens, so high-temperature pyrolysis requires mitigation strategies. The use of catalytic reforming or multi-stage pyrolysis (e.g., recovering tar up to 500 °C and then cracking at higher temperatures) may be possible to balance yield, quality, and environmental safety.

The overall thermal degradation pathways are similar between straw and other biomass types, including willow, but straw exhibits distinct characteristics that cause it to differ from the others. It contains more ash and nitrogen, which catalyze the breakdown of carbohydrates and form unique compounds such as phenyl carbamate and eugenol acetate. In light of the feedstock-specific behavior of lignocellulosic materials during pyrolysis, tailored processing strategies are necessary to optimize the pyrolysis process. Temperature is the master control variable that shapes the chemical profiles of straw-derived volatiles. Chemical analysis, combined with statistical validation, provides a comprehensive understanding of the thermal decomposition dynamics. The insights gained from these studies are crucial for optimizing thermochemical conversion pathways, reducing pollution emissions, and tailoring processes to meet the specific characteristics of feedstocks.

## 5. Conclusions

The pyrolysis of wheat straw exhibits a transparent and predictable pattern of chemical transformation, with temperature being the primary factor that influences the volatile product composition. The volatile fraction below 550 °C consists primarily of oxygenated monomers, such as furans, anhydrosugars (e.g., levoglucosan), and lignin-derived phenolics, including guaiacol and syringol. Approximately 49.6% of volatiles are phenolics, and 27.9% are sugar derivatives. At this point, no polycyclic aromatic hydrocarbons (PAHs) have been detected, in addition to being functionally complex. The bio-oil produced is rich in functional groups and chemically complex; however, due to its high oxygen content and low thermal stability, it requires further processing before it can be used as a fuel.

The volatile profile shifts to simpler phenolic compounds such as phenol, cresols, and catechols at intermediate temperatures, indicating advanced lignin depolymerization. Phenolic content increases as the temperature rises within this window, reaching a maximum near 650 °C and accounting for approximately 60% of the identified volatiles. Around 750 °C, secondary tar formation and deoxygenation processes begin, along with the first PAHs that include naphthalene and fluorene. The chemical composition of tar becomes complex at temperatures between 750 and 850 °C as phenolics and PAHs coexist in roughly equal proportions. Despite the factors such as PAHs gaining 17.8% of the volatile profile, this regime appears to be statistically distinct from that of 550 °C.

Volatiles from above 850 °C are dominated by highly deoxygenated multi-ring aromatic compounds, including PAHs, to the extent that they account for 76.4% of the total. Parallel to this decline, phenolic content, which is decomposed and transformed into more thermally stable polyaromatic compounds, drops sharply to 6.3%. The result is the release of environmental persistence compounds, such as pyrene, fluoranthene, and benz[a]anthracene. Under these conditions, tar becomes more refractory and challenging to condense, resembling a soot precursor rather than a liquid fuel intermediate. The findings demonstrate that while high temperatures are necessary for complete deoxygenation and gas production, toxic tars and PAHs must also be treated downstream, such as by catalytic reforming.

## Data Availability

The original contributions presented in this study are included in the article. Further inquiries can be directed to the corresponding author.
